# Endothelial heterogeneity in the umbilico-placental unit: DNA methylation as an innuendo of epigenetic diversity

**DOI:** 10.3389/fphar.2014.00049

**Published:** 2014-03-27

**Authors:** Paola Casanello, Daniela Schneider, Emilio A. Herrera, Ricardo Uauy, Bernardo J. Krause

**Affiliations:** ^1^Division of Obstetrics and Gynaecology, School of Medicine, Faculty of Medicine, Pontificia Universidad Católica de ChileSantiago, Chile; ^2^Division of Paediatrics, School of Medicine, Pontificia Universidad Católica de ChileSantiago, Chile; ^3^Programa de Fisiopatologïa, Laboratorio de Función y Reactividad Vascular, Instituto de Ciencias Biomédicas, Facultad de Medicina, Universidad de ChileSantiago, Chile

**Keywords:** endothelial, epigenetics, artery, vein, placenta, umbilical

## Abstract

The endothelium is a multifunctional heterogeneous tissue playing a key role in the physiology of every organ. To accomplish this role the endothelium presents a phenotypic diversity that is early prompted during vascular development, allowing it to cope with specific requirements in a time- and site-specific manner. During the last decade several reports show that endothelial diversity is also present in the umbilico-placental vasculature, with differences between macro- and microvascular vessels as well as arterial and venous endothelium. This diversity is evidenced *in vitro* as a higher angiogenic capacity in the microcirculation; or disparity in the levels of several molecules that control endothelial function (i.e., receptor for growth factors, vasoactive mediators, and adhesion molecules) which frequently are differentially expressed between arterial and venous endothelium. Emerging evidence suggests that endothelial diversity would be prominently driven by epigenetic mechanisms which also control the basal expression of endothelial-specific genes. This review outlines evidence for endothelial diversity since early stages of vascular development and how this heterogeneity is expressed in the umbilico-placental vasculature. Furthermore a brief picture of epigenetic mechanisms and their role on endothelial physiology emphasizing new data on umbilical and placental endothelial cells is presented. Unraveling the role of epigenetic mechanisms on long term endothelial physiology and its functional diversity would contribute to develop more accurate therapeutic interventions. Altogether these data show that micro- versus macro-vascular, or artery versus vein comparisons are an oversimplification of the complexity occurring in the endothelium at different levels, and the necessity for the future research to establish the precise source of cells which are under study.

## INTRODUCTION

Since the discovery of the role of endothelium on vascular tone regulation at the beginning of 1980s, a countless number of studies have shown the plethora of remarkable functions that this tissue has in vascular physiology. Notably significant advances in understanding the role of endothelium have used human umbilical and placental vessels as experimental models, which is also applied to the knowledge regarding endothelial diversity. The diversity of functions that the endothelium exerts (i.e., regulation of vessel tone, angiogenesis, immune cell adhesion and migration, exchange, and haemostasis) associates with specific “zones” of the vasculature, suggesting that endothelial cells present a phenotypic heterogeneity that supports this functional diversity (Atkins et al., 2011). From the molecular point of view endothelial cells *in vivo* express several proteins which allow to distinguish between arterial and venous endothelial cells and some of these patterns are preserved *in vitro*, suggesting that long term endothelial physiology is importantly influenced by epigenetic mechanisms (Matouk and Marsden, 2008; Aird, 2012).

### ORIGINS OF ENDOTHELIAL CELLS

Vasculogenesis is the process by which vessels are formed from mesenchymal-derived hemangioblasts which differentiate into endothelial cells (Demir et al., 2007). Current evidence shows that initial stages of vascular development are determined by genetic factors (le Noble et al., 2008; Atkins et al., 2011). These processes require the expression of VEGF (Shalaby et al., 1995) and activation of downstream mitogenic effectors (Parenti et al., 1998; Shizukuda et al., 1999). However, the site from which the vascular progenitors for placental and embryo vasculogenesis emerge is still debated. It is accepted that in the embryo vascular progenitors emerge from intra- and extra-embryonic mesodermal tissues (Jin and Patterson, 2009), whilst in the placenta they arise from the extra-embryonic mesoderm (Chaddha et al., 2004). However, there is growing evidence for a crucial role of the yolk sac in embryo and placental vascular development (Freyer and Renfree, 2009). Indeed, using a sodium-calcium exchanger (*Ncx-1*) knockout mice which fails to initiate cardiac contraction Lux et al. (2008) showed that all the hematopoietic progenitor cells emerge from the yolk sac. The origin of placental endothelial cells could have an important impact on its vascular physiology because arterial-venous identity is early established by environmental cues which could have diverse effects depending on the localization in the embryo.

Growth and consolidation of the placental vascular tree occurs by angiogenesis. In this process single vessels are formed by endothelial precursor cells (EPCs) which differentiate into endothelial cells, and/or proliferate from endothelial cells. These vessels can spread in two ways, (1) non-branching angiogenesis, which implies an increase in the length of the villous vessels, and (2) branching angiogenesis, in which multiple short capillary loops are formed (Demir et al., 2007), increasing the vascular surface area. After these processes have taken place, the vessels mature and their structures stabilize. Additional maturation and specialization in the vascular system are influenced by environmental signals, such as blood flow, oxygen tension, oxidative stress, and epigenetic factors (le Noble et al., 2008; Atkins et al., 2011). All these factors have been implicated in the development and function of the human placenta (Fowden et al., 2008; Burton, 2009; Dennery, 2010). Thus, angiogenesis is a complex process which involves genetic, epigenetic and environmental commands in the development and establishment of the vasculature.

## EPIGENETICS OVERVIEW

During the last decade, the study of genome-environment interactions has revealed a plethora of mechanisms that modulate short and long term cellular physiology. These mechanisms involve mainly epigenetic processes which control chromatin accessibility in a gene- and cell-specific manner. Definition of epigenetics is still under debate mainly due to the several molecular mechanisms that it comprises and the heritability of these changes in an organism and its progeny; however, a simple and broad definition considers epigenetic mechanisms as “chromosome-based mechanisms that change the phenotypic plasticity in a cell or organism” (Krause et al., 2009; Gibney and Nolan, 2010).

**FIGURE 1:**
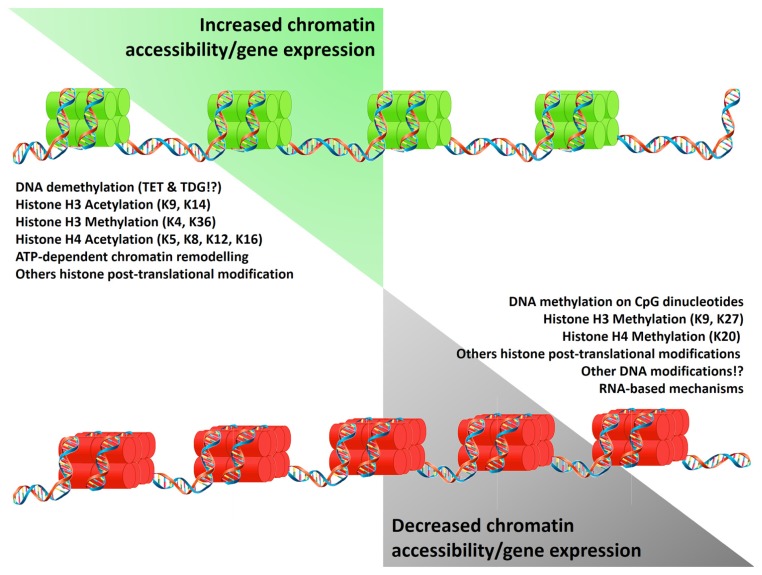
**Epigenetic mechanisms regulating gene expression.** Epigenetic mechanisms control gene expression by increasing (green histones) or limiting chromatin accessibility (red histones). These states result from the equilibrium of modifications on the DNA and histones which reduce (left list) or promote (right list) DNA/histones interactions. Alternatively gene expression is limited by the presence of non-coding RNA. Symbols “!?” denote recently reported mechanisms whose effects are currently under study.

Epigenetic mechanisms affect chromatin structure and gene expression regulating DNA and histone interactions, and the translation and stability of mRNA. Epigenetic markers such as DNA methylation, histone deacetylation, and other repressive histone post-translational modifications (PTMs) alter the structure of the chromatin, generating regions with a “closed chromatin” conformation. Conversely, DNA demethylation (potentially driven by the oxidation of methylated cytosines and their replacement by base excision repair; Kohli and Zhang, 2013), ATP-dependent chromatin remodeling, histone acetylation (Ac), and other permissive histone PTMs, convert the closed chromatin into an “open chromatin” conformation allowing binding of transcription factors and the RNA polymerase II (**Figure 1**). As an additional epigenetic mechanism, the presence of non-coding RNAs can post-transcriptionally repress gene expression. Detailed reviews of the diverse epigenetic mechanisms and their effects on gene expression are available (Klose and Bird, 2006; Kouzarides, 2007; Wang et al., 2007a,b; Kaikkonen et al., 2011; Kohli and Zhang, 2013).

From a developmental perspective epigenetic mechanisms allow the generation of diverse cell phenotypes and functions of an organism from a single genome, and respond to a range of environmental fluctuations. This issue is especially evident in organs and tissues whose structure and function are under constant change across lifespan, such as the cardiovascular system (Aird, 2012). Nonetheless, placental vasculature may also be programmed by epigenetic mechanisms, which are currently under restless research.

### EPIGENETICS IN ENDOTHELIAL PHYSIOLOGY AND PATHOPHYSIOLOGY

Vascular development, endothelial differentiation and function require a fine epigenetic tuning (**Table 1**). Initial steps of vascular development in the embryo seem to be influenced by both genetic and environmental stimuli which drive the emergence of two different populations of endothelial cells (Atkins et al., 2011). Differentiation of embryonic stem cells and EPCs into endothelial cells requires the participation of histone deacetylases (HDAC), lysine demethylases (KDM) and reduced DNA methylation in the promoter region of endothelial-specific genes (Rossig et al., 2005; Zeng et al., 2006; Lagarkova et al., 2008; Banerjee and Bacanamwo, 2010; Ohtani et al., 2011). Conversely, differentiated endothelial cells can be reprogramed to a pseudo-embryonic stem cell phenotype increasing the DNA methylation status of endothelial-specific genes (Lagarkova et al., 2010). In endothelial cells, activating histone PTM, such as acetylation of H3 and H4 and methylation of H3K4, control the basal expression of vWF (Peng and Jahroudi, 2003), NOTCH4 (Wu et al., 2005), VEGF receptor 1 (Dutta et al., 2008), endomucin (Kanki et al., 2011), and eNOS (Fish et al., 2005; Gan et al., 2005).

**Table 1:** Effect of DNA methylation and histone post-translational modifications (PTMs) on endothelial cell physiology.

Mechanism	Process	Reference
DNA methylation	*In vitro* and *in vivo* progenitor endothelial cells differentiatio Activation of tissue-specific genes Ischemia-induced neo-vascularization	Chan et al. (2004), Lagarkova et al. (2008), Ohtani et al. (2011), Rao et al. (2011)
Histone acetylation	Hypoxia-, VEGF- and shear stress- induced angiogenesis VEGF-induced progenitor endothelial cells differentiation Basal endothelial cell-specific genes	Kim et al. (2001), Deroanne et al. (2002), Rossig et al. (2002), Peng and Jahroudi (2003), Illi et al. (2005), Zeng et al. (2006), Wu et al. (2005)
Other histone PTMs	Progenitor endothelial cells differentiation Hypoxia induced eNOS down-regulation	Ohtani et al. (2011), Fish et al. (2010)

On the other hand, HDAC activity is required for an adequate vascular integrity (Chang et al., 2006) preventing short term endothelial proliferation and angiogenesis (Ha et al., 2008; Jin et al., 2011), whilst calmodulin-lysine *N*-methyltransferase (KMT) activity has the opposite effect (Diehl et al., 2007). However, long term HDAC activity promotes angiogenesis in response to VEGF (Deroanne et al., 2002) and hypoxia (Kim et al., 2001) increasing the expression of VEGF (Ruchko et al., 2009) and eNOS (Rossig et al., 2002). Similarly, HDAC activity is increased in response to shear stress (Illi et al., 2003) improving cell survival (Zampetaki et al., 2010) and eNOS expression (Wang et al., 2010). Noteworthy, the epigenetic regulation of NOS3 gene has been extensively studied in endothelial and non-endothelial cells, showing that endothelial cells have a distinctive pattern of DNA methylation and histone PTMs (Fish and Marsden, 2006). Fish et al. (2007) reported that the decreased expression of eNOS in HUVEC exposed to acute hypoxia is controlled by the overexpression of a natural *cis*-antisense non-coding RNA called sONE, and changes in histone PTMs which occur specifically at the eNOS promoter (Fish et al., 2010). Additionally, abrogation of NOS3 promoter DNA methylation increases basal eNOS mRNA expression *in vitro*, and protects against hind-limb ischemic injury *in vivo* (Rao et al., 2011).

Several studies show that epigenetic mechanisms participate in the increased risk of developing vascular diseases. In humans, endothelial cells from atherosclerotic plaques have decreased levels of estrogen receptor β along with increased DNA methylation at the promoter region of this gene, compared with those from non-atherosclerotic plaque regions (Kim et al., 2007). Deficiency of a specific KDM, lysine-specific demethylase-1 (LSD1, KDM1a), associates with decreased expression of eNOS and NO-dependent vasodilation, as well as, salt sensitive hypertension (Pojoga et al., 2011). In newborn rats with persistent pulmonary hypertension, the increased expression of eNOS mRNA is accompanied by augmented levels of acetylated H3 and H4 in the NOS3 gene promoter (Xu et al., 2010). Alternatively, cultured endothelial cells exposed to elevated levels of homocysteine, which relates with increased cardiovascular risk, present decreased proliferation and increased levels of oxidative stress. In both cases homocysteine acts inducing specific hypomethylation of the gene promoters for the cell cycle regulator cyclin A (Jamaluddin et al., 2007) and the pro-oxidant protein p66shc (Kim et al., 2011). Additionally, high glucose-induced endothelial dysfunction requires the participation of HAT (Chen et al., 2010) and KMT (El-Osta et al., 2008), generating important epigenomic changes (Pirola et al., 2011), which can persist several days after the exposure to the noxa (El-Osta et al., 2008).

Notably, vascular physiology is also influenced by epigenetic mechanisms occurring in smooth muscle cells (SMCs). Development of vascular dysfunction is accompanied by changes in SMC phenotype, which shift from a “contractile” to a “synthetic” and “pro-inflammatory” phenotype with long term consequences in the contractile properties of vessels (Owens et al., 2004; Orr et al., 2010). Increasing data shows that this “phenotypic switching” requires the participation of epigenetic mechanisms which establish an altered SMC function (Alexander and Owens, 2012).

## PHENOTYPIC AND EPIGENETIC DIVERSITY IN THE UMBILICO-PLACENTAL ENDOTHELIUM

Pioneer studies by Lang et al. (1993) demonstrated that micro- and macrovascular umbilico-placental endothelium present different immunoreactivity to diverse molecular markers for endothelial cells, suggesting the presence of a phenotypic endothelial diversity in the placenta. Additional evidence from cultured human endothelial cells isolated from the placental microcirculation (PLEC) and the umbilical vein (HUVEC) show that microvascular endothelial cells express higher levels of vascular mediators (angiotensin II, endothelin, and thromboxane; Lang, 2003). Also a differential pattern of homeobox genes (Murthi et al., 2007, 2008) and higher cholesterol transport capacity (Stefulj et al., 2009) in PLEC compared to HUVEC has been shown.

Notably, studies on endothelial cells from arteries and veins have revealed important differences between arterial and venous cells at the same vascular level. In fact the higher mitogenic response observed in PLEC (Lang, 2003) may reflect the combination of a high response to VEGF present in arterial PLEC (PLAEC) and to PIGF in venous endothelial cells (PLVEC; Lang et al., 2008). A transcriptomic analysis between PLAEC and PLVEC showed that they have differential expression of more than 3,000 genes (Lang et al., 2008). Similarly there is a differential expression of eNOS, a key vascular gene, between micro- and macrovascular, and venous and arterial endothelium (Andersen et al., 2009; Krause et al., 2012) being more homogenous at the arterial side (Andersen et al., 2009). This opens the queries about the differences initially reported between micro- and macrovascular endothelium reflecting an endothelial diversity between large and small vessels, and whether they include variances between arteries and veins.

Several studies comparing simultaneously umbilical arterial (HUAEC) and venous (HUVEC) endothelium support the concept that these cells are not a homogenous population, and the necessity of clarifying the precise source of cells when the term “macrovasculature” is used. A general characterization shows that there is a different profile of phospholipids with higher levels of arachidonic acid-related species and heterogeneous expression pattern of selenoproteins (Miller et al., 2002) in HUAEC compared to HUVEC (Takamura et al., 1990). Alongside the classical molecular markers for arterial endothelium, cultured HUAEC express higher levels of PAI 1 (Gallicchio et al., 1994), Cx40 (Van Rijen et al., 1997), 17β-HSD2 (Simard et al., 2011), and VCAM-1 (Egorova et al., 2012); and lower levels of von Willebrand Factor (Shahani et al., 2010) and estrogen receptors beta (ERβ; Simard et al., 2011) compared with HUVEC. On the other hand expressions of pro-constrictive mediators such as angiotensin converting enzyme (Ito et al., 2002) and ET-1 (Egorova et al., 2012) are different in HUVEC relative to HUAEC. Furthermore, expression and activity of eNOS are higher in freshly isolated HUVEC than HUAEC (Andersen et al., 2009) and this expression pattern is also observed in cells cultured up to third passage (Krause et al., 2012). Whether these differences reflects the physiology of umbilical (and potentially placental) arteries and veins, and how they are preserved *in vitro* need further examination. Two recent reports show that the differential gene expression between HUAEC and HUVEC is partially controlled by specific transcription factors. Overexpression of the venous-specific nuclear receptor COUP-TFII in HUAEC decreases the expression of arterial markers (i.e., Hey2, EphrinB2 and NICD4), and its down-regulation in HUVEC increases the expression of arterial markers such as VEGF-A, Dll and EphrinB2 (Korten et al., 2013). Moreover, *in vitro* simultaneous overexpression of eight arterial-specific transcription factors turns the HUVEC transcriptome into a HUAEC-like pattern (Aranguren et al., 2013).

Therefore, the phenotypic diversity in the umbilico-placental circulation is apparently commanded, at least in part, by an equivalent diversity in epigenetic mechanisms.

### ENDOTHELIAL DIVERSITY AND ANGIOGENESIS

In terms of angiogenesis, microvascular endothelial cells present a higher mitogenic response to VEGF, PIGF (Lang, 2003; Lang et al., 2008), and prokineticin 1 (Brouillet et al., 2010) compared with HUVEC, along with an increased expression of pro-angiogenic HOX genes (i.e., TLX1, TLX2, and PHOX1; Murthi et al., 2008). These data are in agreement with the notion that placental angiogenic capacity is augmented in microvascular vessels compared to endothelial cells from larger vessels. However, it is also possible to find significant differences in the angiogenic response in endothelial cells from umbilical arteries and veins. *In vivo* VEGFR3, which is commonly expressed in lymphatic endothelium or during active angiogenesis (Koch and Claesson-Welsh, 2012), is absent in HUAEC but expressed in HUVEC (Veikkola et al., 2003). Moreover *in vitro* chemotaxis induced by VEGFA or FGF2 is higher in HUVEC compared to HUAEC (Barkefors et al., 2008), and netrin-1 prevents the VEGF-induced migration in HUAEC without effect on HUVEC (Lu et al., 2004). Further studies are needed to address the effects and the role on placental physiology of this increased angiogenic response observed in HUVEC.

### ENDOTHELIAL DIVERSITY IN RESPONSE TO STRESS

Placental vascular and endothelial physiology, similar to adult vasculature, are importantly influenced by stimuli such as altered shear stress and oxygen levels whose effects are apparently different between arteries and veins. Normally arterial endothelium is exposed to higher shear stress and therefore it is plausible to predict a stronger response to increasing stress. In fact pulsatile shear stress increases the expression of arterial markers (i.e., Hey1, Hey1, and ephrinB2) in HUAEC but decreases the expression of venous markers (COUP-TFII) in HUVEC (Buschmann et al., 2010). Laminar shear stress have similar effects on the expression of arterial-venous markers in these cells, and increases the levels of *S*-nitrosylated proteins (Hoffmann et al., 2003) endothelin-1, VCAM, and vWF (Egorova et al., 2012) in HUAEC compared to HUVEC. Whether these differences are observed in microvascular endothelial cells remains to be determined.

Some evidence regarding the effects of low oxygen levels on endothelial function in placental large and small vessels, as well as arteries and veins, show a differential vascular response to hypoxia throughout the placenta (Krause et al., 2011, 2012). On the other hand placental endothelium is importantly exposed to low oxygen levels and oxidative stress which are negative regulator of placental angiogenesis (Burton et al., 2009). A reduction in oxygen levels from 21 to 12% O_2_decreases placental venous microvascular endothelial cells viability with no effect on their arterial counterparts (Lassance et al., 2012), and PLAEC exposed to 3% O_2_ show an increased mitogenic response to VEGFA and FGF2 compared to cells cultured at 21% O_2_ (Wang et al., 2009). This higher response to VEGFA and FGF2 is also observed in HUAEC exposed to physiological levels of oxygen (3–5% O_2_; Jiang et al., 2013). Additionally, hypoxia (1% O_2_) increases the expression of the pro-angiogenic factor protease-activated receptor 2 in HUVEC and this effect is higher in HUAEC (Svensson et al., 2011).

**FIGURE 2:**
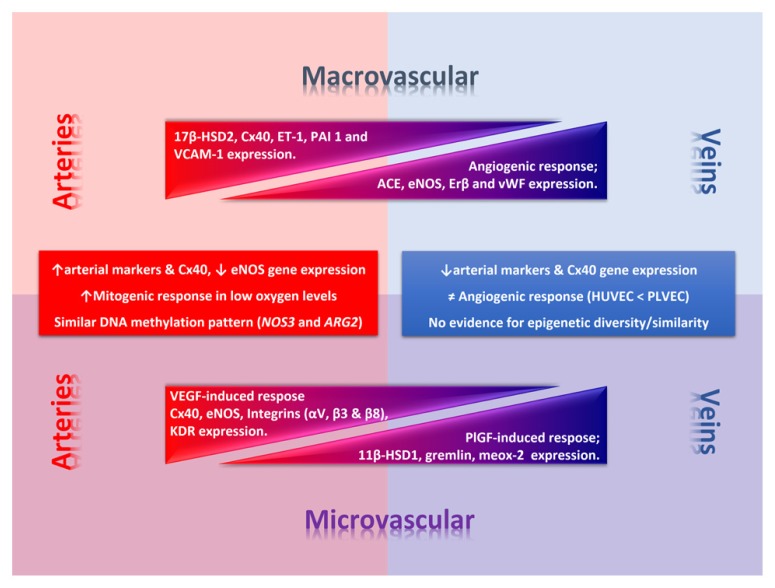
**Phenotypic diversity in the umbilico-placental endothelium.** Umbilical (macrovascular) and placental (microvascular) endothelial cells present a phenotypic diversity characterized by a differential response to angiogenic factors and gene expression of key endothelial genes. In the figure thickness of the triangle denotes deferential relative expression (or response) regarding arteries or veins at each level. Square text box includes common traits between macro- and micro-vascular arterial or venous endothelial cells. 11β-HSD1, 11β-hydroxysteroid dehydrogenase 1; 17β-HSD2, 17β hydroxysteroid dehydrogenase 2; ARG2, arginase-2 gene; Cx40, conexin-40; eNOS, endothelial nitric oxide synthase; ET-1, endothelin-1; KDR, vascular endothelial growth factor (VEGF) receptor 2; NOS3, eNOS gene; PAI 1, plasminogen activator inhibitor-1; PlGF, placental growth factor; VCAM-1, vascular cell adhesion molecule 1.

Altogether these data show that venous-arterial endothelial phenotypic diversity occurs among umbilical and placental vessels (**Figure 2**). Further studies should include control comparison between arterial and venous endothelial cells from the same branching level to rule out potential differences attributable to arteries and veins rather than micro- and macrovascular vessels. It is worth to note that most of the differences occurring among these cells types could be reverted by genetic manipulation. However, its persistence *in vitro* suggests that additional mechanisms controlling gene expression should be operating, arguing for a crucial role for epigenetics in this process.

### EPIGENETIC AND PLACENTAL ENDOTHELIAL DIVERSITY

Compelling evidence shows the fundamental role of epigenetics controlling the endothelial-specific gene expression, however, the next frontier is to determine how epigenetic mechanisms influence the endothelial functional diversity. Two recent reports studying placental and umbilical endothelial cells suggest the presence of significant differences in the DNA methylation of gene promoters which could be responsible for the differential gene expression present in these cells.

A comparison of the genome-wide DNA methylation profile in PLAEC and PLVEC show that venous endothelial cells present lower levels of global methylation compared to PLAEC (Joo et al., 2013) which could reflect the immature phenotype of PLVEC (Lang et al., 2008). Further analysis show the presence of several genes which are differentially methylated between PLAEC and PLVEC, and some of them present an inverse correlation between the level of methylation and the gene expression. Notably those genes are considered endothelial markers and play a key role in vascular physiology, such as eNOS, vWF, Conexin40, VEGFR1, VEGFC, and angiopiotein-1. However, there are endothelial genes whose promoters do not present any correlation between methylation levels and gene expression, such as VEGFR2, Hey2, NOTCH, EphB2, and EphB4 (Joo et al., 2013).

Conversely, the comparison of DNA methylation status of NOS3 (eNOS) and ARG2 (arginase-2) promoters by pyrosequencing in HUAEC, PLAEC and HUVEC, suggest the presence of site-specific differences between these cells. Methylation status at NOS3 promoter in umbilical and placental endothelial cells showed differences in three specific CpG between arterial and venous endothelial cells (Krause et al., 2013). Two of these differentially methylated CpGs correspond to the reported hypoxia response element (-5369 and -5375; Coulet et al., 2003) which regulates the response to hypoxia and show lower methylation levels in PLAEC and HUAEC compared to HUVEC. Whether this variation participates in the differential regulation of eNOS expression in response to hypoxia that has been reported between HUAEC and HUVEC (Krause et al., 2012) needs to be addressed. An additional differentially methylated CpG is located at -352 from the transcription starting site, showing a higher methylation pattern in arterial relative to venous cells. Moreover the methylation status at this CpG suggests an inverse correlation between DNA methylation and eNOS expression, which is higher in HUVEC (lower methylation levels) compared to HUAEC (Krause et al., 2012, 2013). It is also reported that CpG -352 is differentially methylated between HUVEC and human dermal microvascular endothelial cells (Chan et al., 2004), having the later a methylation status comparable to that found in HUAEC and PLAEC, which suggest that CpG -352 might play a role in the differential regulation of basal eNOS expression in arterial and venous endothelial cells. Krause et al. (2013) compared the NOS3 promoter DNA methylation status between control and endothelial cells isolated from pregnancies with intrauterine growth restriction (IUGR). Remarkably changes in DNA methylation in IUGR cells are restricted to those CpGs that are differentially methylated in normal endothelial cells. In fact, IUGR HUAEC and PLAEC present similar changes at CpGs -5375 (increased methylation) and -352 (decreased methylation) compared with normal cells, and these methylation levels are comparable to that find in normal HUVEC. Conversely, changes in the DNA methylation status of IUGR HUVEC where at CpGs -5369 (decreased methylation) and -352 (increased methylation), and they are comparable to those find in normal HUAEC and PLAEC. The methylation levels at CpG -352 in IUGR HUAEC and HUVEC are also related with the levels of mRNA for eNOS (Krause et al., 2013), reinforcing the potential importance of CpG -352 in the regulation of basal eNOS expression. Finally analysis of methylation status of ARG2 promoter in HUAEC, PLAEC, and HUVEC show a single difference between PLAEC and HUVEC, however, it is still unknown if there is a correlation with arginase-2 expression and activity.

DNA methylation is one of the main epigenetic mechanisms that controls long term gene expression, showing a high reproducibility after every cellular replication and this characteristic is driven by the activity of DNA methyltransferase-1 (DNMT1). In IUGR HUAEC and HUVEC DNMT1 silencing shows a differential effect, reducing and increasing basal eNOS expression, respectively (Krause et al., 2013). Silencing of DNMT1 restores to normal eNOS mRNA levels in IUGR HUAEC and HUVEC, and this effect is not observed on arginase-2 expression where it further increases its expression in IUGR HUVEC, without any effect in IUGR HUAEC (Krause et al., 2013). This suggests that DNA methylation (Jamaluddin et al., 2007; Banerjee and Bacanamwo, 2010; Kim et al., 2011) and other epigenetic mechanisms (Kim et al., 2001; Deroanne et al., 2002; Fish et al., 2010) control gene expression in endothelial cells in a gene-specific manner.

Although the studies in PLAEC and PLVEC (Joo et al., 2013), and in HUAEC and HUVEC (Krause et al., 2013) used two different approaches to analyze the DNA methylation patterns, there are some similarities in the outcomes. First, both studies show that methylation status of NOS3 proximal promoter inversely correlates with the levels of mRNA for eNOS, and this occurs in cells exposed for several days to culture conditions. Second, PLAEC and PLVEC show differential levels arginase-2 expression without differences in the DNA methylation in ARG2 promoter, whilst in control and IUGR HUAEC differences in DNA methylation are not associated to difference in arginase-2 expression. Finally, DNMT1 silencing in IUGR cells normalize eNOS expression but not arginase-2 expression.

## CONCLUSION

Altogether these seminal data show that epigenetic mechanisms could be responsible for the phenotypic diversity of endothelial cells in the umbilico-placental unit, and these mechanisms would be operating in a cell- and gene-specific manner. The current research on the area is offering novel data about potential mechanisms but still further studies are required to have a comprehensive picture of the additional epigenetic mechanisms controlling the gene expression in physiological and pathophysiological conditions and its consequences in umbilico-placental functions.

## Conflict of Interest Statement

The authors declare that the research was conducted in the absence of any commercial or financial relationships that could be construed as a potential conflict of interest.

## References

[B1] Aird W. C. (2012). Endothelial cell heterogeneity. *Cold Spring Harb. Perspect. Med. * 2:a006429 10.1101/cshperspect.a006429 PMC3253027 22315715

[B2] Alexander M. R. Owens G. K. (2012). Epigenetic control of smooth muscle cell differentiation and phenotypic switching in vascular development and disease. *Annu. Rev. Physiol.* 74 13–40 10.1146/annurev-physiol-012110-142315 22017177

[B3] Andersen M. R. Simonsen U. Uldbjerg N. Aalkjaer C. Stender S. (2009). Smoking cessation early in pregnancy and birth weight, length, head circumference, and endothelial nitric oxide synthase activity in umbilical and chorionic vessels: an observational study of healthy singleton pregnancies. *Circulation* 119 857–864 10.1161/CIRCULATIONAHA.107.755769 19188513

[B4] Aranguren X. L. Agirre X. Beerens M. Coppiello G. Uriz M. Vandersmissen I. (2013). Unraveling a novel transcription factor code determining the human arterial-specific endothelial cell signature. *Blood* 122 3982–3992 10.1182/blood-2013-02-483255 24108462

[B5] Atkins G. B. Jain M. K. Hamik A. (2011). Endothelial differentiation: molecular mechanisms of specification and heterogeneity. *Arterioscler. Thromb. Vasc. Biol.* 31 1476–1484 10.1161/ATVBAHA.111.228999 21677290 PMC3134408

[B6] Banerjee S. Bacanamwo M. (2010). DNA methyltransferase inhibition induces mouse embryonic stem cell differentiation into endothelial cells. *Exp. Cell Res.* 316 172–180 10.1016/j.yexcr.2009.08.011 19715692 PMC2788076

[B7] Barkefors I. Le Jan S. Jakobsson L. Hejll E. Carlson G. Johansson H. (2008). Endothelial cell migration in stable gradients of vascular endothelial growth factor A and fibroblast growth factor 2: effects on chemotaxis and chemokinesis. *J. Biol. Chem.* 283 13905–13912 10.1074/jbc.M704917200 18347025

[B8] Brouillet S. Hoffmann P. Benharouga M. Salomon A. Schaal J. P. Feige J. J. (2010). Molecular characterization of EG-VEGF-mediated angiogenesis: differential effects on microvascular and macrovascular endothelial cells. *Mol. Biol. Cell* 21 2832–2843 10.1091/mbc.E10-01-0059 PMC2921113 20587779

[B9] Burton G. J. (2009). Oxygen, the Janus gas; its effects on human placental development and function. *J. Anat.* 215 27–35 10.1111/j.1469-7580.2008.00978.x 19175804 PMC2714636

[B10] Burton G. J. Charnock-Jones D. S. Jauniaux E. (2009). Regulation of vascular growth and function in the human placenta. *Reproduction* 138 895–902 10.1530/REP-09-0092 19470597

[B11] Buschmann I. Pries A. Styp-Rekowska B. Hillmeister P. Loufrani L. Henrion D. (2010). Pulsatile shear and Gja5 modulate arterial identity and remodeling events during flow-driven arteriogenesis. *Development* 137 2187–2196 10.1242/dev.045351 20530546

[B12] Chaddha V. Viero S. Huppertz B. Kingdom J. (2004). Developmental biology of the placenta and the origins of placental insufficiency. *Semin. Fetal Neonatal Med.* 9 357–369 10.1016/j.siny.2004.03.006 15691771

[B13] Chan Y. Fish J. E. D’abreo C. Lin S. Robb G. B. Teichert A. M. (2004). The cell-specific expression of endothelial nitric-oxide synthase: a role for DNA methylation. *J. Biol. Chem.* 279 35087–35100 10.1074/jbc.M405063200 15180995

[B14] Chang S. Young B. D. Li S. Qi X. Richardson J. A. Olson E. N. (2006). Histone deacetylase 7 maintains vascular integrity by repressing matrix metalloproteinase 10. *Cell* 126 321–334 10.1016/j.cell.2006.05.040 16873063

[B15] Chen S. Feng B. George B. Chakrabarti R. Chen M. Chakrabarti S. (2010). Transcriptional coactivator p300 regulates glucose-induced gene expression in endothelial cells. *Am. J. Physiol. Endocrinol. Metab.* 298 E127–E137 10.1152/ajpendo.00432.2009 19903865

[B16] Coulet F. Nadaud S. Agrapart M. Soubrier F. (2003). Identification of hypoxia-response element in the human endothelial nitric-oxide synthase gene promoter. *J. Biol. Chem.* 278 46230–46240 10.1074/jbc.M305420200 12963737

[B17] Demir R. Seval Y. Huppertz B. (2007). Vasculogenesis and angiogenesis in the early human placenta. *Acta Histochem.* 109 257–265 10.1016/j.acthis.2007.02.008 17574656

[B18] Dennery P. A. (2010). Oxidative stress in development: nature or nurture? *Free Radic. Biol. Med.* 49 1147–1151 10.1016/j.freeradbiomed.2010.07.011 20656021

[B19] Deroanne C. F. Bonjean K. Servotte S. Devy L. Colige A. Clausse N. (2002). Histone deacetylases inhibitors as anti-angiogenic agents altering vascular endothelial growth factor signaling. *Oncogene* 21 427–436 10.1038/sj.onc.1205108 11821955

[B20] Diehl F. Rossig L. Zeiher A. M. Dimmeler S. Urbich C. (2007). The histone methyltransferase MLL is an upstream regulator of endothelial-cell sprout formation. *Blood* 109 1472–1478 10.1182/blood-2006-08-039651 17047146

[B21] Dutta D. Ray S. Vivian J. L. Paul S. (2008). Activation of the VEGFR1 chromatin domain: an angiogenic signal-ETS1/HIF-2alpha regulatory axis. *J. Biol. Chem.* 283 25404–25413 10.1074/jbc.M804349200% 18625704 PMC2533069

[B22] Egorova A. D. Deruiter M. C. De Boer H. C. Van De Pas S. Gittenberger-De Groot A. C. Van Zonneveld A. J. (2012). Endothelial colony-forming cells show a mature transcriptional response to shear stress. *In Vitro Cell. Dev. Biol. Anim.* 48 21–29 10.1007/s11626-011-9470-z% 22101679

[B23] El-Osta A. Brasacchio D. Yao D. Pocai A. Jones P. L. Roeder R. G. (2008). Transient high glucose causes persistent epigenetic changes and altered gene expression during subsequent normoglycemia. *J. Exp. Med.* 205 2409–2417 10.1084/jem.20081188 18809715 PMC2556800

[B24] Fish J. E. Marsden P. A. (2006). Endothelial nitric oxide synthase: insight into cell-specific gene regulation in the vascular endothelium. *Cell. Mol. Life Sci.* 63 144–162 10.1007/s00018-005-5421-8 16416260 PMC11136399

[B25] Fish J. E. Matouk C. C. Rachlis A. Lin S. Tai S. C. D’abreo C. (2005). The expression of endothelial nitric-oxide synthase is controlled by a cell-specific histone code. *J. Biol. Chem.* 280 24824–24838 10.1074/jbc.M502115200 15870070

[B26] Fish J. E. Matouk C. C. Yeboah E. Bevan S. C. Khan M. Patil K. (2007). Hypoxia-inducible expression of a natural cis-antisense transcript inhibits endothelial nitric-oxide synthase. *J. Biol. Chem.* 282 15652–15666 10.1074/jbc.M608318200 17403686

[B27] Fish J. E. Yan M. S. Matouk C. C. St Bernard R. Ho J. J. Gavryushova A. (2010). Hypoxic repression of endothelial nitric-oxide synthase transcription is coupled with eviction of promoter histones. *J. Biol. Chem.* 285 810–826 10.1074/jbc.M109.067868 19880524 PMC2801283

[B28] Fowden A. L. Forhead A. J. Coan P. M. Burton G. J. (2008). The placenta and intrauterine programming. *J. Neuroendocrinol.* 20 439–450 10.1111/j.1365-2826.2008.01663.x 18266944

[B29] Freyer C. Renfree M. B. (2009). The mammalian yolk sac placenta. *J. Exp. Zool. B Mol. Dev. Evol.* 312 545–554 10.1002/jez.b.21239 18985616

[B30] Gallicchio M. Argyriou S. Ianches G. Filonzi E. L. Zoellner H. Hamilton J. A. (1994). Stimulation of PAI-1 expression in endothelial cells by cultured vascular smooth muscle cells. *Arterioscler. Thromb.* 14 815–823 10.1161/01.ATV.14.5.815 8172858

[B31] Gan Y. Shen Y. H. Wang J. Wang X. Utama B. Wang X. L. (2005). Role of histone deacetylation in cell-specific expression of endothelial nitric-oxide synthase. *J. Biol. Chem.* 280 16467–16475 10.1074/jbc.M412960200 15722551 PMC1283144

[B32] Gibney E. R. Nolan C. M. (2010). Epigenetics and gene expression. *Heredity *(*Edinb.*) 105 4–13 10.1038/hdy.2010.54 20461105

[B33] Ha C. H. Jhun B. S. Kao H. Y. Jin Z. G. (2008). VEGF stimulates HDAC7 phosphorylation and cytoplasmic accumulation modulating matrix metalloproteinase expression and angiogenesis. *Arterioscler. Thromb. Vasc. Biol.* 28 1782–1788 10.1161/ATVBAHA.108.172528 18617643 PMC2746922

[B34] Hoffmann J. Dimmeler S. Haendeler J. (2003). Shear stress increases the amount of S-nitrosylated molecules in endothelial cells: important role for signal transduction. *FEBS Lett.* 551 153–158 10.1016/S0014-5793(03)00917-7 12965221

[B35] Illi B. Nanni S. Scopece A. Farsetti A. Biglioli P. Capogrossi M. C. (2003). Shear stress-mediated chromatin remodeling provides molecular basis for flow-dependent regulation of gene expression. *Circ. Res.* 93 155–161 10.1161/01.RES.0000080933.82105.29 12805238

[B36] Illi B. Scopece A. Nanni S. Farsetti A. Morgante L. Biglioli P. (2005). Epigenetic histone modification and cardiovascular lineage programming in mouse embryonic stem cells exposed to laminar shear stress. *Circ. Res.* 96 501–508 10.1161/01.RES.0000159181.06379.63. 15705964

[B37] Ito M. Itakura A. Ohno Y. Nomura M. Senga T. Nagasaka T. (2002). Possible activation of the renin-angiotensin system in the feto-placental unit in preeclampsia. *J. Clin. Endocrinol. Metab.* 87 1871–1878 10.1210/jcem.87.4.8422 11932332

[B38] Jamaluddin M. D. Chen I. Yang F. Jiang X. Jan M. Liu X. (2007). Homocysteine inhibits endothelial cell growth via DNA hypomethylation of the cyclin A gene. *Blood* 110 3648–3655 10.1182/blood-2007-06-096701 17698632 PMC2077313

[B39] Jiang Y. Z. Wang K. Li Y. Dai C. F. Wang P. Kendziorski C. (2013). Enhanced cellular responses and distinct gene profiles in human fetoplacental artery endothelial cells under chronic low oxygen. *Biol. Reprod.* 89 133 10.1095/biolreprod.113.110551 PMC4076354 24152727

[B40] Jin G. Bausch D. Knightly T. Liu Z. Li Y. Liu B. (2011). Histone deacetylase inhibitors enhance endothelial cell sprouting angiogenesis in vitro. *Surgery* 150 429–435 10.1016/j.surg.2011.07.001 21878227 PMC3164968

[B41] Jin S. W. Patterson C. (2009). The opening act: vasculogenesis and the origins of circulation. *Arterioscler. Thromb. Vasc. Biol.* 29 623–629 10.1161/ATVBAHA.107.161539 19008532 PMC3432309

[B42] Joo J. E. Hiden U. Lassance L. Gordon L. Martino D. J. Desoye G. (2013). Variable promoter methylation contributes to differential expression of key genes in human placenta-derived venous and arterial endothelial cells. *BMC Genomics * 14:475 10.1186/1471-2164-14-475 PMC3729658 23855827

[B43] Kaikkonen M. U. Lam M. T. Glass C. K. (2011). Non-coding RNAs as regulators of gene expression and epigenetics. *Cardiovasc. Res.* 90 430–440 10.1093/cvr/cvr097 21558279 PMC3096308

[B44] Kanki Y. Kohro T. Jiang S. Tsutsumi S. Mimura I. Suehiro J. (2011). Epigenetically coordinated GATA2 binding is necessary for endothelium-specific endomucin expression. *EMBO J.* 30 2582–2595 10.1038/emboj.2011.173 21666600 PMC3155306

[B45] Kim C. S. Kim Y. R. Naqvi A. Kumar S. Hoffman T. A. Jung S. B. (2011). Homocysteine promotes human endothelial cell dysfunction via site-specific epigenetic regulation of p66shc. *Cardiovasc. Res.* 92 466–475 10.1093/cvr/cvr250 21933910 PMC3211975

[B46] Kim J. Kim J. Y. Song K. S. Lee Y. H. Seo J. S. Jelinek J. (2007). Epigenetic changes in estrogen receptor beta gene in atherosclerotic cardiovascular tissues and in-vitro vascular senescence. *Biochim. Biophys. Acta* 1772 72–80 10.1016/j.bbadis.2006.10.004 17110088

[B47] Kim M. S. Kwon H. J. Lee Y. M. Baek J. H. Jang J. E. Lee S. W. (2001). Histone deacetylases induce angiogenesis by negative regulation of tumor suppressor genes. *Nat. Med.* 7 437–443 10.1038/86507 11283670

[B48] Klose R. J. Bird A. P. (2006). Genomic DNA methylation: the mark and its mediators. *Trends Biochem. Sci.* 31 89–97 10.1016/j.tibs.2005.12.008 16403636

[B49] Koch S. Claesson-Welsh L. (2012). Signal transduction by vascular endothelial growth factor receptors. *Cold Spring Harb. Perspect. Med. * 2:a006502 10.1101/cshperspect.a006502 PMC3385940 22762016

[B50] Kohli R. M. Zhang Y. (2013). TET enzymes, TDG and the dynamics of DNA demethylation. *Nature* 502 472–479 10.1038/nature12750 24153300 PMC4046508

[B51] Korten S. Brunssen C. Poitz D. M. Grossklaus S. Brux M. Schnittler H. J. (2013). Impact of Hey2 and COUP-TFII on genes involved in arteriovenous differentiation in primary human arterial and venous endothelial cells. *Basic Res. Cardiol.* 108 362 10.1007/s00395-013-0362-0. 23744056

[B52] Kouzarides T. (2007). Chromatin modifications and their function. *Cell* 128 693–705 10.1016/j.cell.2007.02.005 17320507

[B53] Krause B. Sobrevia L. Casanello P. (2009). Epigenetics: new concepts of old phenomena in vascular physiology. *Curr. Vasc. Pharmacol.* 7 513–520 10.2174/157016109789043883 19485890

[B54] Krause B. J. Costello P. M. Munoz-Urrutia E. Lillycrop K. A. Hanson M. A. Casanello P. (2013). Role of DNA methyltransferase 1 on the altered eNOS expression in human umbilical endothelium from intrauterine growth restricted fetuses. *Epigenetics* 8 944–952 10.4161/epi.25579 23867713 PMC3883771

[B55] Krause B. J. Hanson M. A. Casanello P. (2011). Role of nitric oxide in placental vascular development and function. *Placenta * 32:797–805 10.1016/j.placenta.2011.06.025 21798594 PMC3218217

[B56] Krause B. J. Prieto C. P. Munoz-Urrutia E. Martin S. S. Sobrevia L. Casanello P. (2012). Role of arginase-2 and eNOS in the differential vascular reactivity and hypoxia-induced endothelial response in umbilical arteries and veins. *Placenta * 33:360–366 10.1016/j.placenta.2012.02.006 22391327

[B57] Lagarkova M. A. Shutova M. V. Bogomazova A. N. Vassina E. M. Glazov E. A. Zhang P. (2010). Induction of pluripotency in human endothelial cells resets epigenetic profile on genome scale. *Cell Cycle* 9 937–946 10.4161/cc.9.5.10869 20160486

[B58] Lagarkova M. A. Volchkov P. Y. Philonenko E. S. Kiselev S. L. (2008). Efficient differentiation of hESCs into endothelial cells in vitro is secured by epigenetic changes. *Cell Cycle* 7 2929–2935 10.4161/cc.7.18.6700 18814342

[B59] Lang I. (2003). Heterogeneity of microvascular endothelial cells isolated from human term placenta and macrovascular umbilical vein endothelial cells. *Eur. J. Cell Biol.* 82 163–173 10.1078/0171-9335-00306 12751902

[B60] Lang I. Hartmann M. Blaschitz A. Dohr G. Skofitsch G. Desoye G. (1993). Immunohistochemical evidence for the heterogeneity of maternal and fetal vascular endothelial cells in human full-term placenta. *Cell Tissue Res.* 274 211–218 10.1007/BF00318740 7505718

[B61] Lang I. Schweizer A. Hiden U. Ghaffari-Tabrizi N. Hagendorfer G. Bilban M. (2008). Human fetal placental endothelial cells have a mature arterial and a juvenile venous phenotype with adipogenic and osteogenic differentiation potential. *Differentiation* 76 1031–1043 10.1111/j.1432-0436.2008.00302.x 18673379

[B62] Lassance L. Miedl H. Konya V. Heinemann A. Ebner B. Hackl H. (2012). Differential response of arterial and venous endothelial cells to extracellular matrix is modulated by oxygen. *Histochem. Cell Biol.* 10.1007/s00418-012-0917-4 [Epub ahead of print] 22294260

[B63] le Noble F. Klein C. Tintu A. Pries A. Buschmann I. (2008). Neural guidance molecules, tip cells, and mechanical factors in vascular development. *Cardiovasc. Res.* 78 232–241 10.1093/cvr/cvn058 18316324

[B64] Lu X. Le Noble F. Yuan L. Jiang Q. De Lafarge B. Sugiyama D. (2004). The netrin receptor UNC5B mediates guidance events controlling morphogenesis of the vascular system. *Nature* 432 179–186 10.1038/nature03080 15510105

[B65] Lux C. T. Yoshimoto M. Mcgrath K. Conway S. J. Palis J. Yoder M. C. (2008). All primitive and definitive hematopoietic progenitor cells emerging before E10 in the mouse embryo are products of the yolk sac. *Blood* 111 3435–3438 10.1182/blood-2007-08-107086 17932251 PMC2275011

[B66] Matouk C. C. Marsden P. A. (2008). Epigenetic regulation of vascular endothelial gene expression. *Circ. Res.* 102 873–887 10.1161/CIRCRESAHA.107.171025 18436802

[B67] Miller S. Walker S. W. Arthur J. R. Lewin M. H. Pickard K. Nicol F. (2002). Selenoprotein expression in endothelial cells from different human vasculature and species. *Biochim. Biophys. Acta* 1588 85–93 10.1016/S0925-4439(02)00143-6 12379318

[B68] Murthi P. Hiden U. Rajaraman G. Liu H. Borg A. J. Coombes F. (2008). Novel homeobox genes are differentially expressed in placental microvascular endothelial cells compared with macrovascular cells. *Placenta* 29 624–630 10.1016/j.placenta.2008.04.006 18514308

[B69] Murthi P. So M. Gude N. M. Doherty V. L. Brennecke S. P. Kalionis B. (2007). Homeobox genes are differentially expressed in macrovascular human umbilical vein endothelial cells and microvascular placental endothelial cells. *Placenta* 28 219–223 10.1016/j.placenta.2006.02.012 16647116

[B70] Ohtani K. Vlachojannis G. J. Koyanagi M. Boeckel J. N. Urbich C. Farcas R. (2011). Epigenetic regulation of endothelial lineage committed genes in pro-angiogenic hematopoietic and endothelial progenitor cells. *Circ. Res.* 109 1219–1229 10.1161/CIRCRESAHA.111.247304 21980126

[B71] Orr A. W. Hastings N. E. Blackman B. R. Wamhoff B. R. (2010). Complex regulation and function of the inflammatory smooth muscle cell phenotype in atherosclerosis. *J. Vasc. Res.* 47 168–180 10.1159/000250095 19851078 PMC2842170

[B72] Owens G. K. Kumar M. S. Wamhoff B. R. (2004). Molecular regulation of vascular smooth muscle cell differentiation in development and disease. *Physiol. Rev.* 84 767–801 10.1152/physrev.00041.2003. 15269336

[B73] Parenti A. Morbidelli L. Cui X. L. Douglas J. G. Hood J. D. Granger H. J. (1998). Nitric oxide is an upstream signal of vascular endothelial growth factor-induced extracellular signal-regulated kinase1/2 activation in postcapillary endothelium. *J. Biol. Chem.* 273 4220–4226 10.1074/jbc.273.7.4220 9461619

[B74] Peng Y. Jahroudi N. (2003). The NFY transcription factor inhibits von Willebrand factor promoter activation in non-endothelial cells through recruitment of histone deacetylases. *J. Biol. Chem.* 278 8385–8394 10.1074/jbc.M213156200 12511565

[B75] Pirola L. Balcerczyk A. Tothill R. W. Haviv I. Kaspi A. Lunke S. (2011). Genome-wide analysis distinguishes hyperglycemia regulated epigenetic signatures of primary vascular cells. *Genome Res.* 21 1601–1615 10.1101/gr.116095.110 21890681 PMC3202278

[B76] Pojoga L. H. Williams J. S. Yao T. M. Kumar A. Raffetto J. D. Do Nascimento G. R. (2011). Histone demethylase LSD1 deficiency during high-salt diet is associated with enhanced vascular contraction, altered NO-cGMP relaxation pathway, and hypertension. *Am. J. Physiol. Heart Circ. Physiol.* 301 H1862–H1871 10.1152/ajpheart.00513.2011 21873498 PMC3213961

[B77] Rao X. Zhong J. Zhang S. Zhang Y. Yu Q. Yang P. (2011). Loss of methyl-CpG-binding domain protein 2 enhances endothelial angiogenesis and protects mice against hind-limb ischemic injury. *Circulation* 123 2964–2974 10.1161/CIRCULATIONAHA.110.966408 21670230 PMC4120778

[B78] Rossig L. Li H. Fisslthaler B. Urbich C. Fleming I. Forstermann U. (2002). Inhibitors of histone deacetylation downregulate the expression of endothelial nitric oxide synthase and compromise endothelial cell function in vasorelaxation and angiogenesis. *Circ. Res.* 91 837–844 10.1161/01.RES.0000037983.07158.B1 12411399

[B79] Rossig L. Urbich C. Bruhl T. Dernbach E. Heeschen C. Chavakis E. (2005). Histone deacetylase activity is essential for the expression of HoxA9 and for endothelial commitment of progenitor cells. *J. Exp. Med.* 201 1825–1835 10.1084/jem.20042097 15928198 PMC2213253

[B80] Ruchko M. V. Gorodnya O. M. Pastukh V. M. Swiger B. M. Middleton N. S. Wilson G. L. (2009). Hypoxia-induced oxidative base modifications in the VEGF hypoxia-response element are associated with transcriptionally active nucleosomes. *Free Radic. Biol. Med.* 46 352–359 10.1016/j.freeradbiomed.2008.09.038 18992807 PMC2645035

[B81] Shahani T. Lavend’homme R. Luttun A. Saint-Remy J. M. Peerlinck K. Jacquemin M. (2010). Activation of human endothelial cells from specific vascular beds induces the release of a FVIII storage pool. *Blood* 115 4902–4909 10.1182/blood-2009-07-232546 20351306

[B82] Shalaby F. Rossant J. Yamaguchi T. P. Gertsenstein M. Wu X. F. Breitman M. L. (1995). Failure of blood-island formation and vasculogenesis in Flk-1-deficient mice. *Nature* 376 62–66 10.1038/376062a0 7596435

[B83] Shizukuda Y. Tang S. Yokota R. Ware J. A. (1999). Vascular endothelial growth factor-induced endothelial cell migration and proliferation depend on a nitric oxide-mediated decrease in protein kinase Cdelta activity. *Circ. Res.* 85 247–256 10.1161/01.RES.85.3.247 10436167

[B84] Simard M. Drolet R. Blomquist C. H. Tremblay Y. (2011). Human type 2 17beta-hydroxysteroid dehydrogenase in umbilical vein and artery endothelial cells: differential inactivation of sex steroids according to the vessel type. *Endocrine* 40 203–211 10.1007/s12020-011-9519-5 21877158

[B85] Stefulj J. Panzenboeck U. Becker T. Hirschmugl B. Schweinzer C. Lang I. (2009). Human endothelial cells of the placental barrier efficiently deliver cholesterol to the fetal circulation via ABCA1 and ABCG1. *Circ. Res.* 104 600–608 10.1161/CIRCRESAHA.108.185066 19168441

[B86] Svensson K. J. Kucharzewska P. Christianson H. C. Skold S. Lofstedt T. Johansson M. C. (2011). Hypoxia triggers a proangiogenic pathway involving cancer cell microvesicles and PAR-2-mediated heparin-binding EGF signaling in endothelial cells. *Proc. Natl. Acad. Sci. U.S.A.* 108 13147–13152 10.1073/pnas.1104261108 21788507 PMC3156184

[B87] Takamura H. Kasai H. Arita H. Kito M. (1990). Phospholipid molecular species in human umbilical artery and vein endothelial cells. *J. Lipid Res.* 31 709–717 2351875

[B88] Van Rijen H. Van Kempen M. J. Analbers L. J. Rook M. B. Van Ginneken A. C. Gros D. (1997). Gap junctions in human umbilical cord endothelial cells contain multiple connexins. *Am. J. Physiol.* 272 C117–C130 9038818 10.1152/ajpcell.1997.272.1.C117

[B89] Veikkola T. Lohela M. Ikenberg K. Makinen T. Korff T. Saaristo A. (2003). Intrinsic versus microenvironmental regulation of lymphatic endothelial cell phenotype and function. *FASEB J.* 17 2006–2013 10.1096/fj.03-0179com 14597670

[B90] Wang G. G. Allis C. D. Chi P. (2007a). Chromatin remodeling and cancer, Part I: covalent histone modifications. *Trends Mol. Medic.* 13 363–372 10.1016/j.molmed.2007.07.003 17822958

[B91] Wang G. G. Allis C. D. Chi P. (2007b). Chromatin remodeling and cancer, Part II: ATP-dependent chromatin remodeling. *Trends Mol. Med.* 13 373–380 10.1016/j.molmed.2007.07.004 17822959 PMC4337864

[B92] Wang K. Jiang Y. Z. Chen D. B. Zheng J. (2009). Hypoxia enhances FGF2- and VEGF-stimulated human placental artery endothelial cell proliferation: roles of MEK1/2/ERK1/2 and PI3K/AKT1 pathways. *Placenta* 30 1045–1051 10.1016/j.placenta.2009.10.007 19892399 PMC2788063

[B93] Wang W. Ha C. H. Jhun B. S. Wong C. Jain M. K. Jin Z. G. (2010). Fluid shear stress stimulates phosphorylation-dependent nuclear export of HDAC5 and mediates expression of KLF2 and eNOS. *Blood* 115 2971–2979 10.1182/blood-2009-05-224824 20042720 PMC2854437

[B94] Wu J. Iwata F. Grass J. A. Osborne C. S. Elnitski L. Fraser P. (2005). Molecular determinants of NOTCH4 transcription in vascular endothelium. *Mol. Cell. Biol.* 25 1458–1474 10.1128/MCB.25.4.1458-1474.2005 15684396 PMC548019

[B95] Xu X. F. Ma X. L. Shen Z. Wu X. L. Cheng F. Du L. Z. (2010). Epigenetic regulation of the endothelial nitric oxide synthase gene in persistent pulmonary hypertension of the newborn rat. *J. Hypertens.* 28 2227–2235 10.1097/HJH.0b013e32833e08f1 20724942

[B96] Zampetaki A. Zeng L. Margariti A. Xiao Q. Li H. Zhang Z. (2010). Histone deacetylase 3 is critical in endothelial survival and atherosclerosis development in response to disturbed flow. *Circulation* 121 132–142 10.1161/CIRCULATIONAHA.109.890491 20026773

[B97] Zeng L. Xiao Q. Margariti A. Zhang Z. Zampetaki A. Patel S. (2006). HDAC3 is crucial in shear- and VEGF-induced stem cell differentiation toward endothelial cells. *J .Cell Biol.* 174 1059–1069 10.1083/jcb.200605113 16982804 PMC2064396

